# Supplementary material for: Shear force effect of the dry process on cathode contact coverage in all-solid-state batteries
Source: Nat Commun. 2024 Jun 4;15:4763. doi: 10.1038/s41467-024-49183-3 (PMC11150245; doi:10.1038/s41467-024-49183-3)
Supplement: Supplementary file 1 — Supplementary Info [file 41467_2024_49183_MOESM1_ESM.pdf]

Supplementary Information

Shear Force Effect of the Dry Process on Cathode Contact Coverage in All-Solid-State Batteries

Dongkyu Lee[1], Yejin Shim[1,2], Youngsung Kim[3], Guhan Kwon[3], Seung Ho Choi[2,*], KyungSu Kim[2,*], and Dong-Joo Yoo[1,*]

[1]School of Mechanical Engineering, Korea University, 145 Anam-ro, Seongbuk-gu, Seoul 02841, Republic of Korea

[2]Advanced Batteries Research Center, Korea Electronics Technology Institute, 25 Saenari-ro, Seongnam 13509, Republic of Korea

[3]Production engineering Research Institute, LG Electronics Incorporation, 10, Magokjungang 10-ro, Gangseo-gu, Seoul, 07796, Republic of Korea

*Corresponding author. E-mail: sh.choi@keti.re.kr, kimkyungsu@keti.re.kr, djyoo@korea.ac.kr

Supplementary Note

The model is comprised of a several domains and boundaries: the electrolyte domain, the electrode domain, the porous conductive binder domain, followed by the internal electrode surface boundary and the electrode surface boundary. In each domain, there are governing equations related to current density and/or ion transport. In the electrolyte, electrode, and porous binder domains, the current densities $(\mathbf{i}_e, \mathbf{i}_c,\ and\ \mathbf{i}_b)$ are governed by the ohm's law:

$$\mathbf{i}_e = -\sigma_e \nabla \phi_e, \tag{1}$$

$$\mathbf{i}_c = -\sigma_c \nabla \phi_c, \tag{2}$$

$$\mathbf{i}_{\mathrm{be}} = -\sigma_{\mathrm{be,eff}} \nabla \phi_e, \tag{3}$$

$$\mathbf{i}_b = -\sigma_{b,\mathrm{eff}} \nabla \phi_b, \tag{4}$$

where σ_e is the ionic conductivity of the electrolyte, ϕ_e is the potential of the electrolyte phase, σ_c is the electrical conductivity of the cathode, and ϕ_c the potential of the cathode. $\sigma_{\mathrm{be,eff}}$ and $\sigma_{b,\mathrm{eff}}$ are the effective ion conductivity and the effective electrical conductivity of the binder domain, and ϕ_e and ϕ_b are the potentials of electrolyte and binder, respectively.

The ion transport inside the cathode active material is computed in the transport of diluted species interface. The ion transport mechanism applied in the model is concentration gradient driven diffusion, explained by the Fick's law. The following equation shows governing equation.

$$\mathbf{J}_s = -D_s \nabla c_s, \tag{5}$$

where $\mathbf{J}_s$ is the lithium-ion flux, D_s is the diffusion coefficient of the solid phase particle, and c_s is the lithium-ion concentration in the particle.

The potential of the cathode is decided by SOC dependent equilibrium potential curve obtained empirically by experiments. [1] SOC is defined as

$$\mathrm{SOC} = \frac{c_s}{c_{\mathrm{max}}}, \tag{6}$$

where c_{max} is the maximum amount of lithium-ion concentration that the active material can hold.

The internal electrode surface (cathode surface) and the electrode surface (anode) both govern the interfacial electrochemical reactions. The electrode kinetics for the electrode surfaces is defined by the Butler-Volmer equation:

$$i = i_0 \left(\exp\left(\frac{\alpha_a F \eta}{RT}\right) - \exp\left(\frac{-\alpha_c F \eta}{RT}\right) \right), \tag{7}$$

where i is the current density, i_0 is the exchange current density, $\alpha_{a,c}$ is the anodic/cathodic transfer coefficient, F is the faraday constant, R is the gas constant, T is the temperature, and η is the overpotential.

Additionally, the double-layer capacitance of the is defined as

$$i_{\mathrm{dl}} = \left(\frac{\partial(\phi_c - \phi_e)}{\partial t}\right) C_{\mathrm{dl}}, \tag{8}$$

where i_{dl} is the current density of the double-layer capacitance, and C_{dl} is the electrical double-layer capacitance.

There are three types of boundary conditions in this model: the insulation, and the charge-discharge cycling in the battery interface and the no flux condition in the transport of diluted species interface. The insulation and the no flux boundary conditions stop current and mass flux. The equation for each boundary conditions are

$$\boldsymbol{i} \cdot \boldsymbol{n} = 0, \tag{9}$$

$$-\boldsymbol{n} \cdot (-D\nabla c) = 0, \tag{10}$$

where $\boldsymbol{i}$ is the current density vector and $\boldsymbol{n}$ is the normal direction vector on boundaries.

The charge-discharge cycling boundary condition applies current onto the positive electrode boundary, defined as

$$\int \boldsymbol{i_c} \cdot \boldsymbol{n}\, dl = I_{\mathrm{cell}}. \tag{11}$$

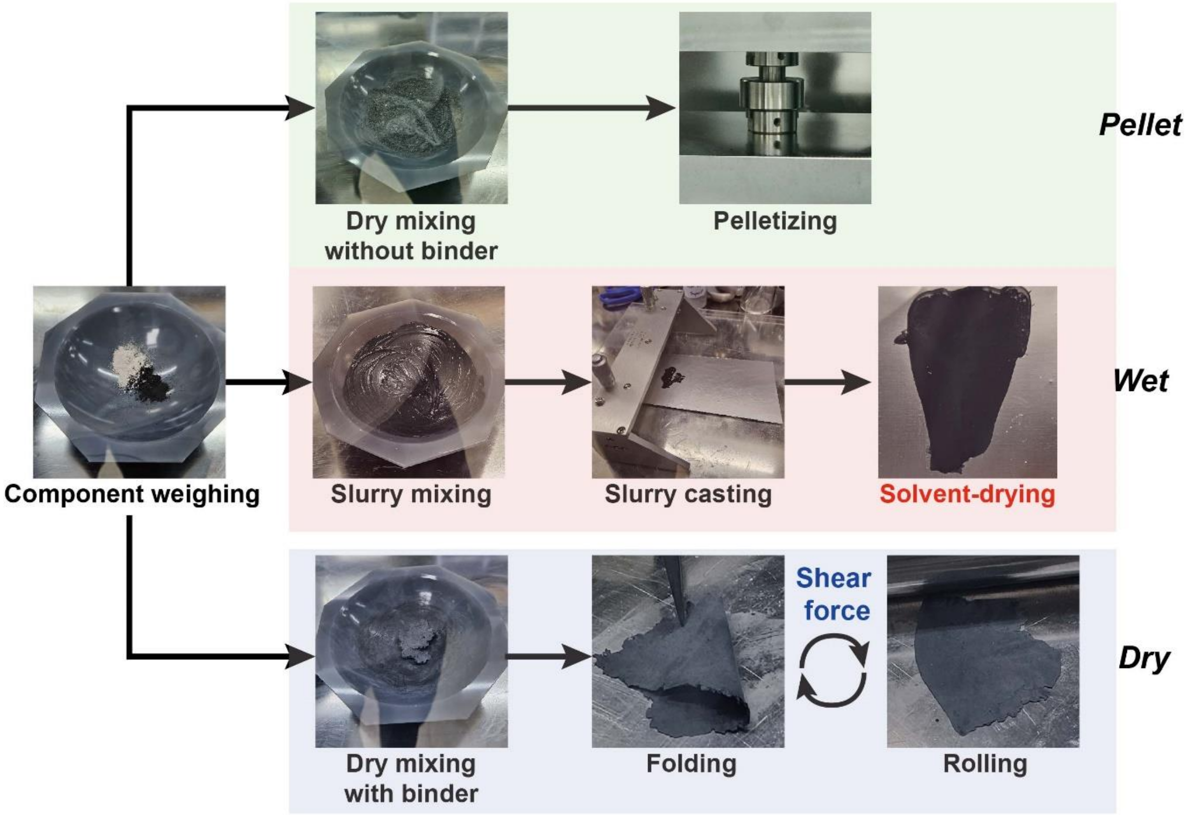

Figure S1. Process for pellet, wet, and dry electrodes.

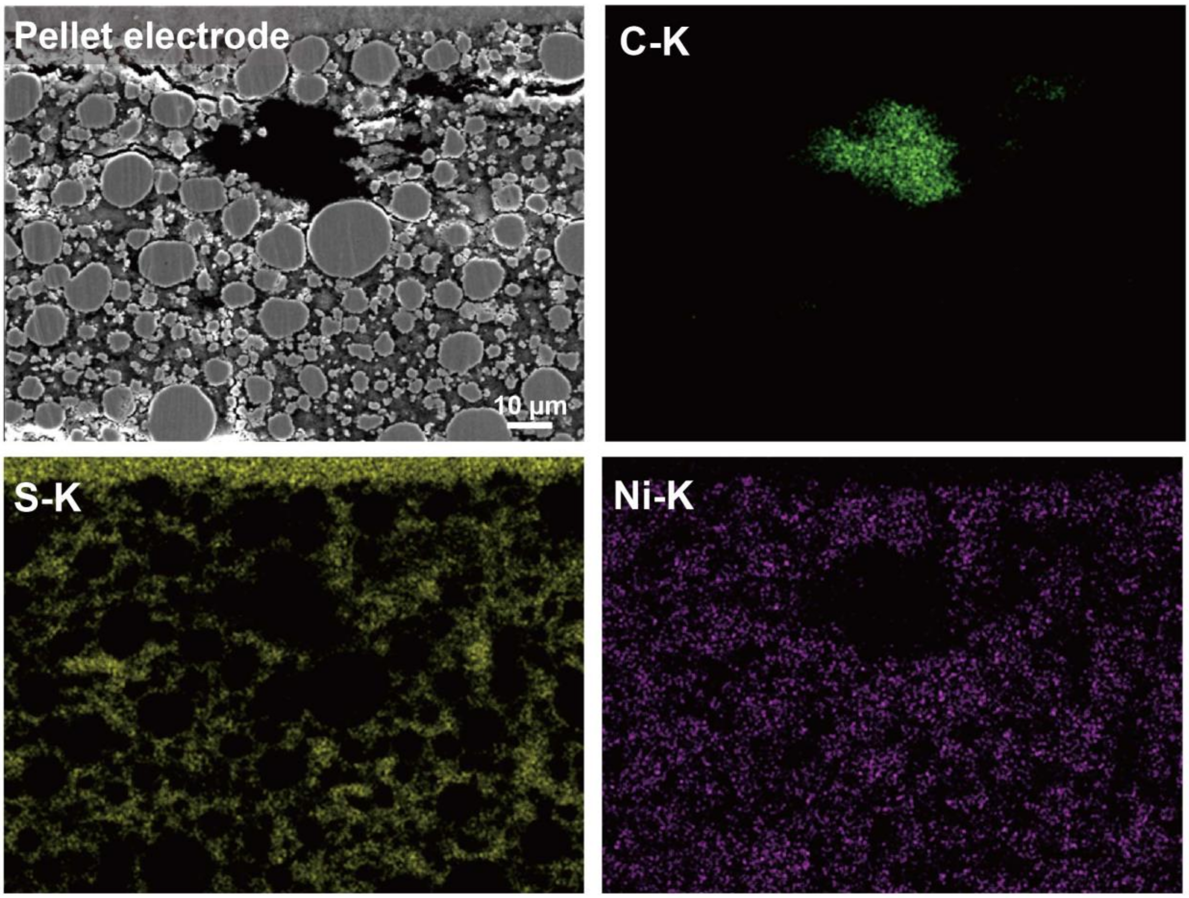

Figure S2. Characterization of pellet electrode. Cross-sectional scanning electron microscope (SEM) image and energy-dispersive X-ray spectroscopy (EDS) mapping in C-K, S-K and Ni-K of pellet electrodes.

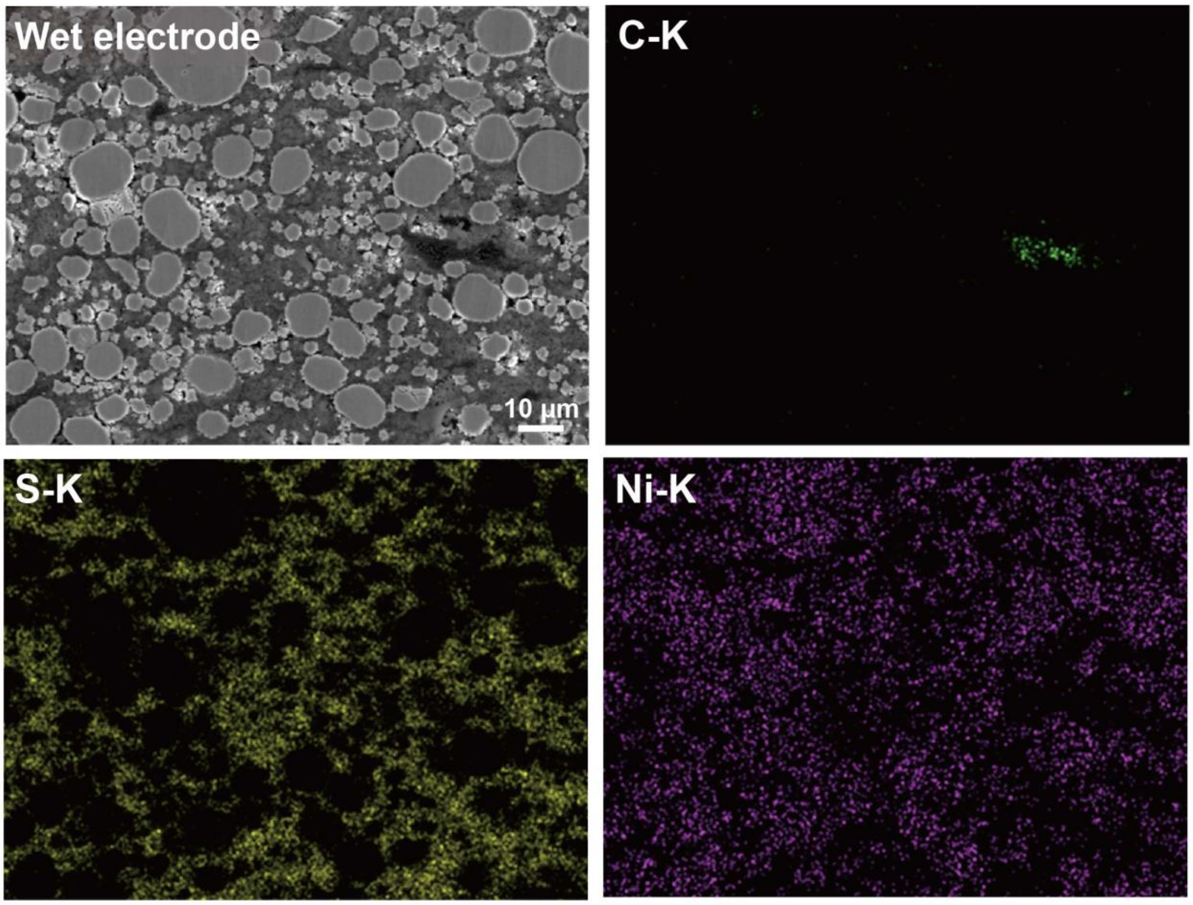

Figure S3. Characterization of wet electrode. Cross-sectional scanning electron microscope (SEM) image and energy-dispersive X-ray spectroscopy (EDS) mapping in C-K, S-K and Ni-K of wet electrodes.

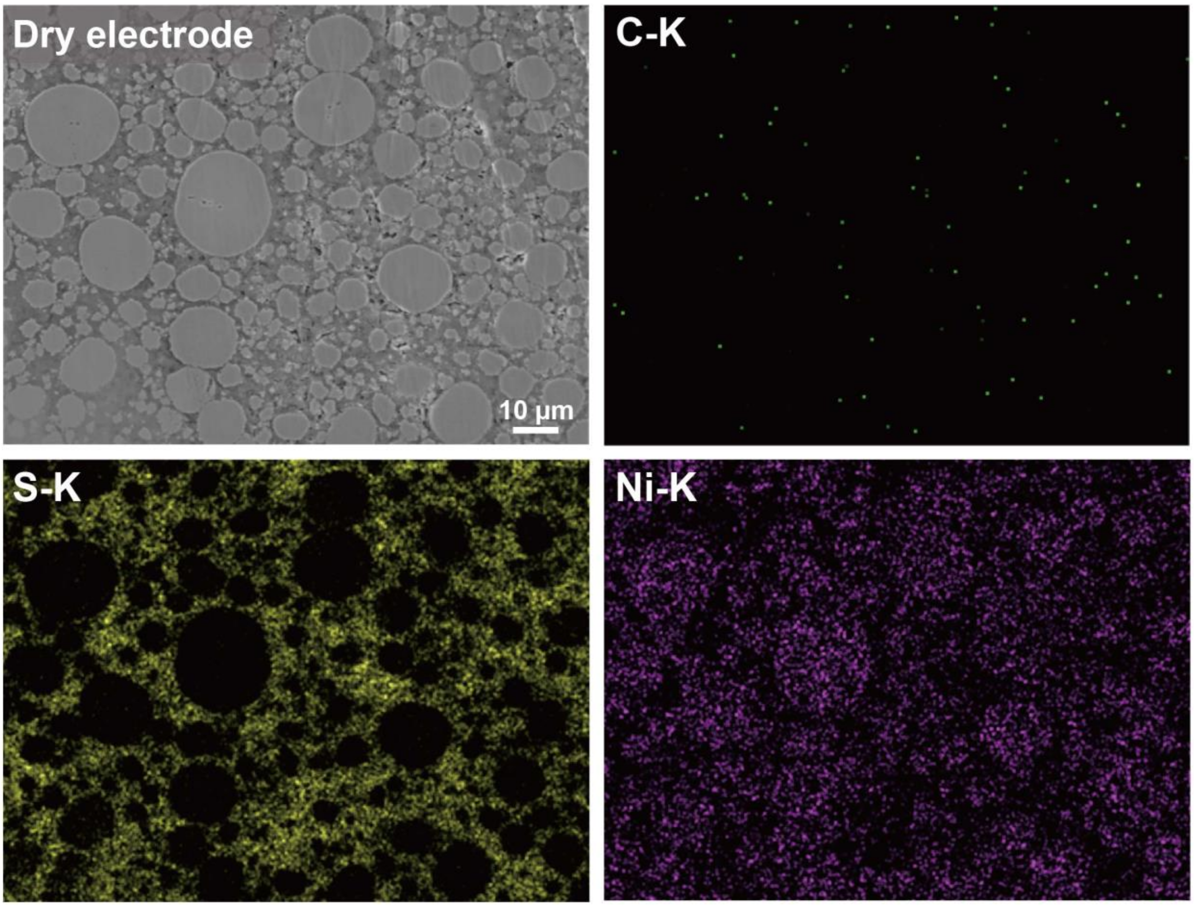

Figure S4. Characterization of dry electrode. Cross-sectional scanning electron microscope (SEM) image and energy-dispersive X-ray spectroscopy (EDS) mapping in C-K, S-K and Ni-K of dry electrodes.

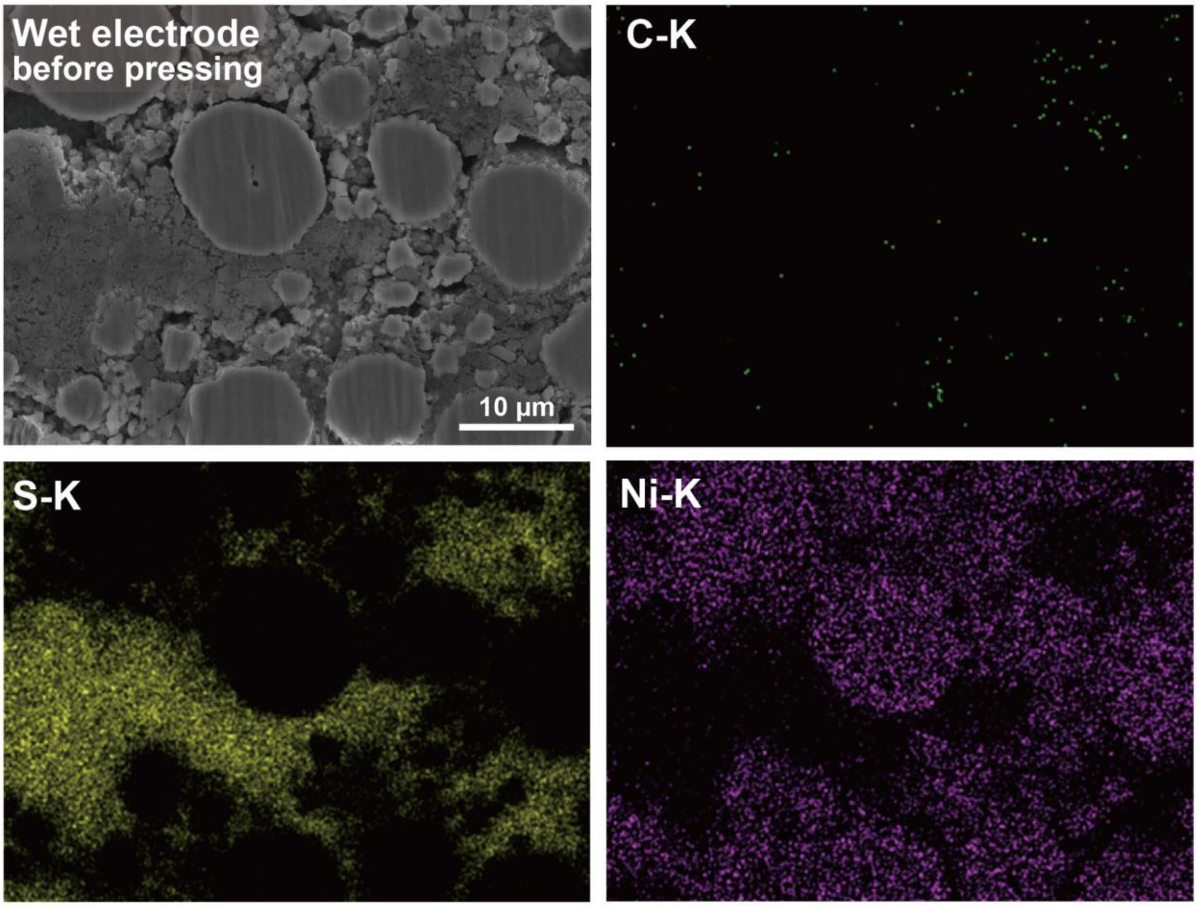

Figure S5. Characterization of wet electrode before pressing. Cross-sectional scanning electron microscope (SEM) image and energy-dispersive X-ray spectroscopy (EDS) mapping in C-K, S-K and Ni-K of wet electrodes before pressing.

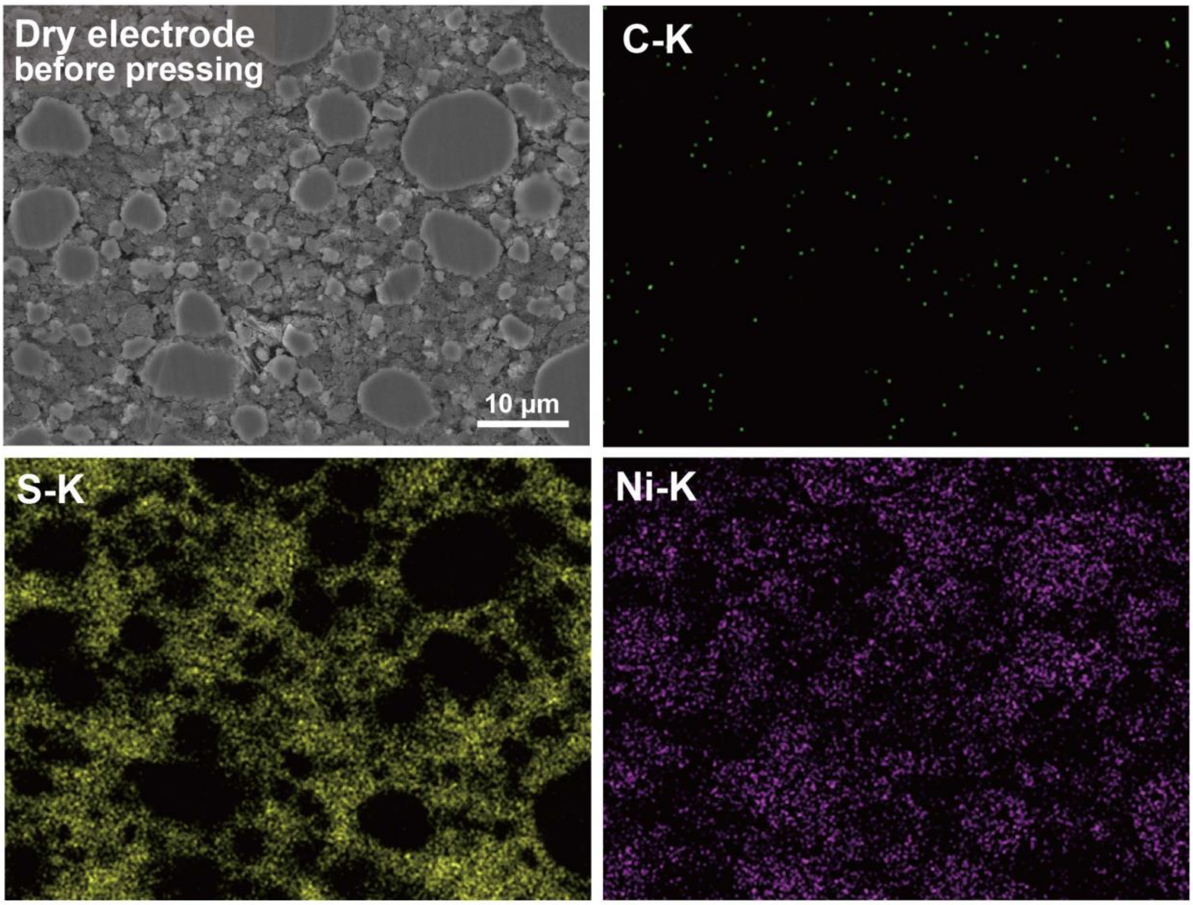

Figure S6. Characterization of dry electrode before pressing. Cross-sectional scanning electron microscope (SEM) image and energy-dispersive X-ray spectroscopy (EDS) mapping in C-K, S-K and Ni-K of dry electrodes before pressing.

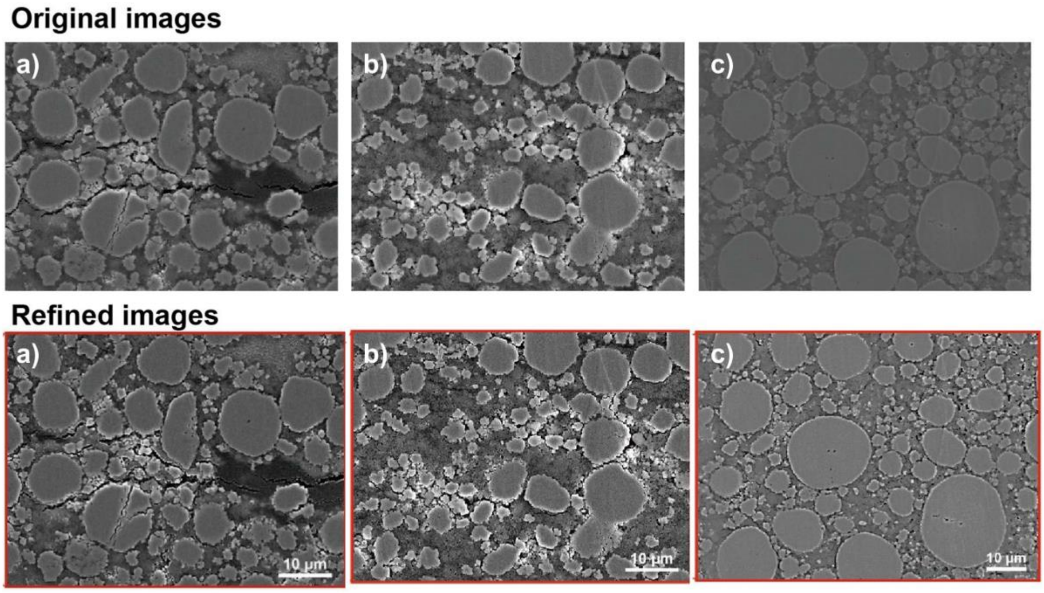

Figure S7. Image refinements. Original and refined scanning electron microscope (SEM) images of (a) pellet, (b) wet-processed, and (c) dry-processed electrodes.

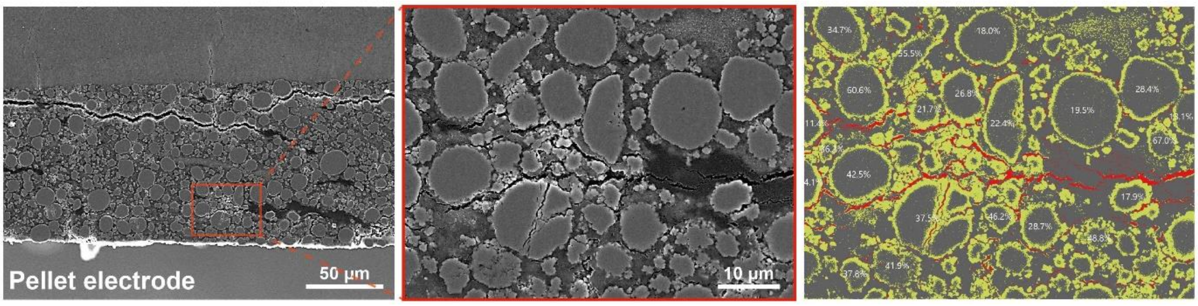

Figure S8. Coverage extraction of pellet electrode. Cross-sectional scanning electron microscope (SEM) images of pellet electrodes.

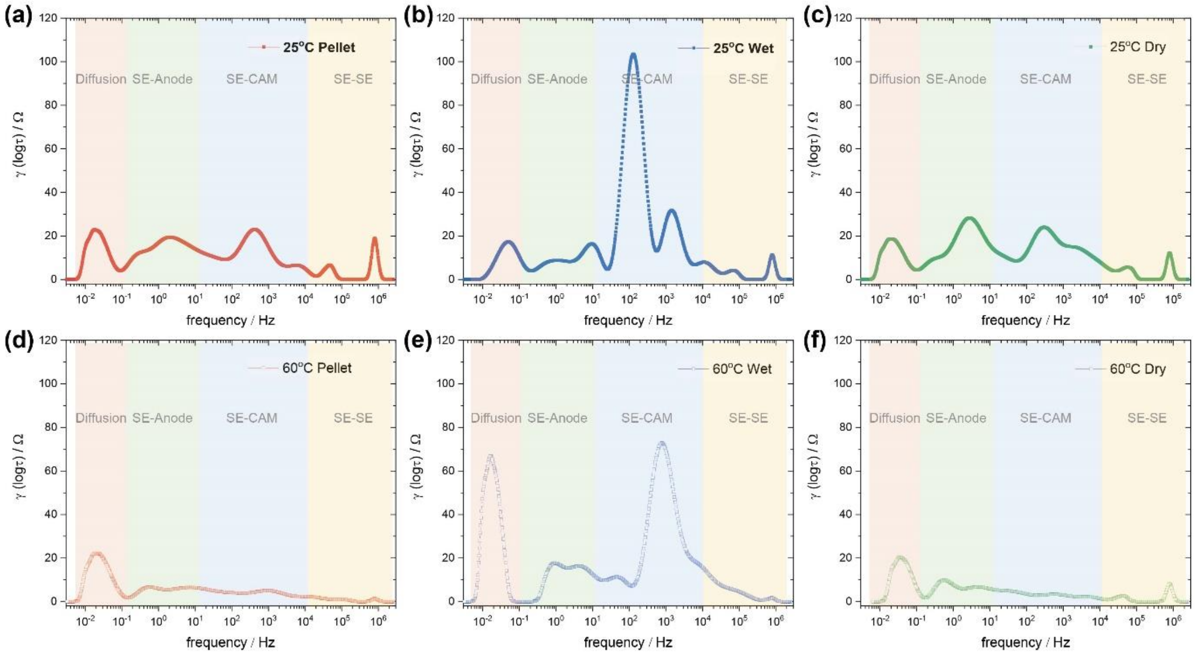

Figure S9. Resistance analysis of electrode configurations. Distribution of relaxation time (DRT) profiles of (a, d) pellet, (b, e) wet-processed, and (c ,f) dry-processed electrodes at 25ºC and 60ºC.

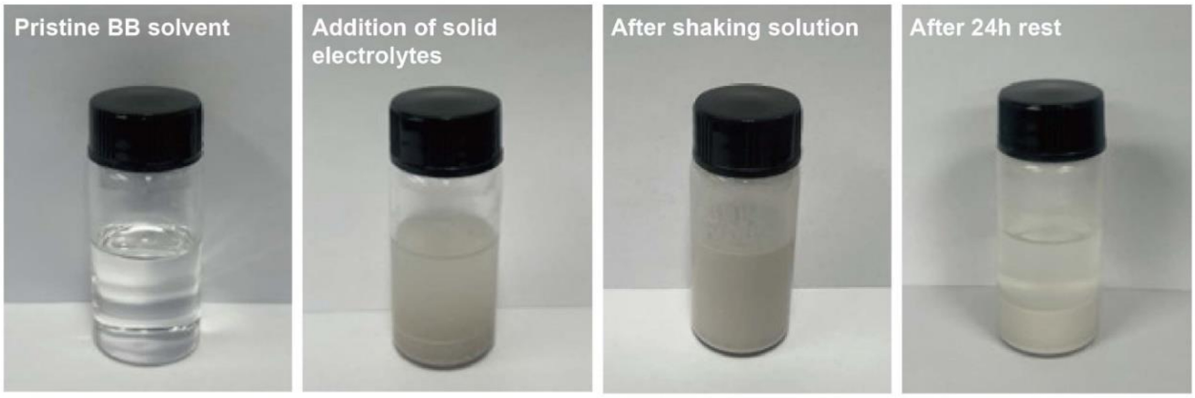

Figure S10. Chemical stability of solid electrolytes and solvents. Optical images of pristine butyl butyrate solvent, after adding solid electrolytes, after shaking solution, and after 24 hours of rest.

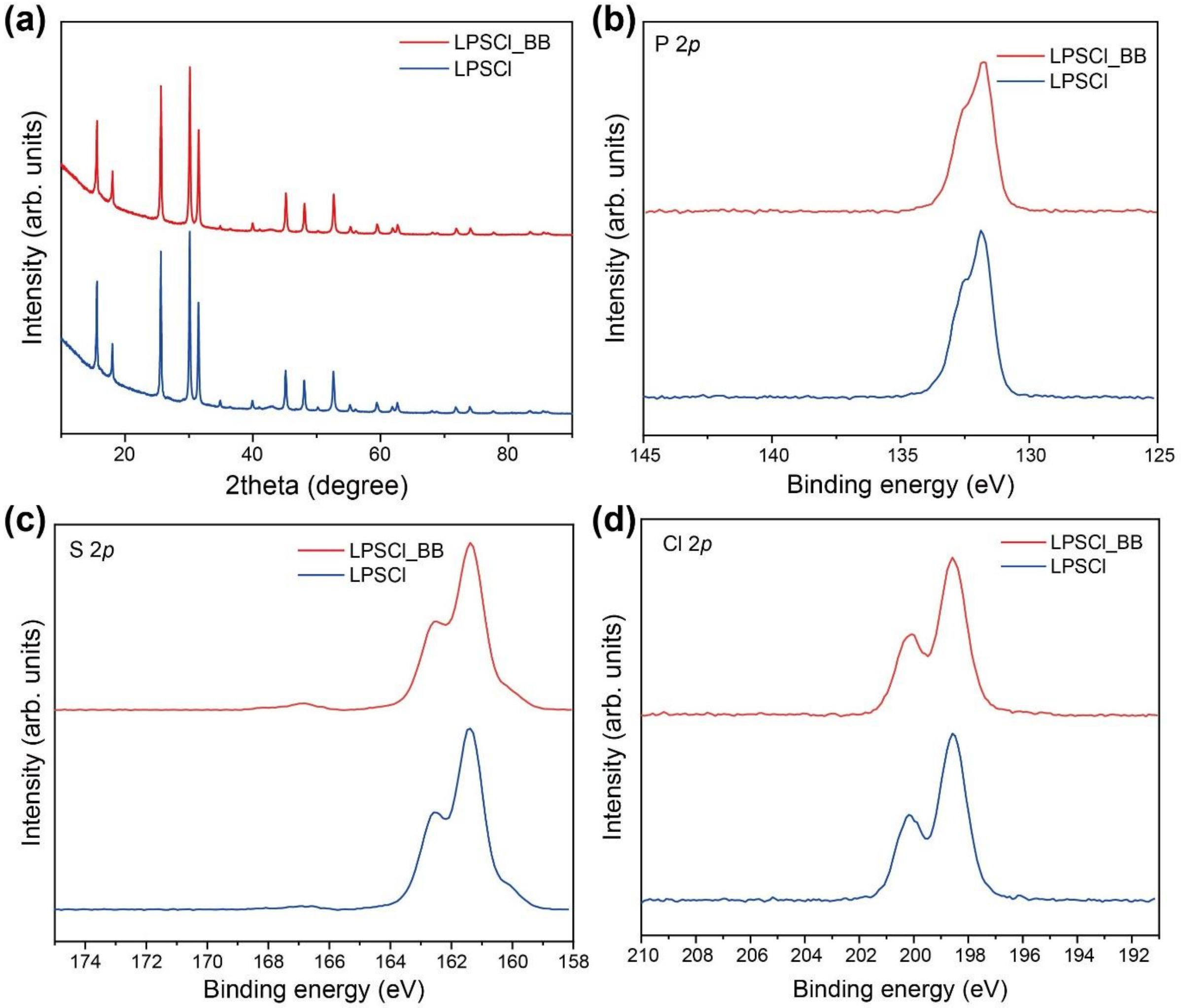

Figure S11. Stability analysis of solid electrolytes and solvents. (a) X-ray diffraction (XRD) spectra and X-ray photoelectron spectroscopy (XPS) spectra of pristine Li_6PS_5Cl (LPSCl) and LPSCl after exposure to butyl butyrate solvent (denoted as LPSCl_BB). (b) P 2*p*, (c) S 2*p*, and (d) Cl 2*p*.

(a)

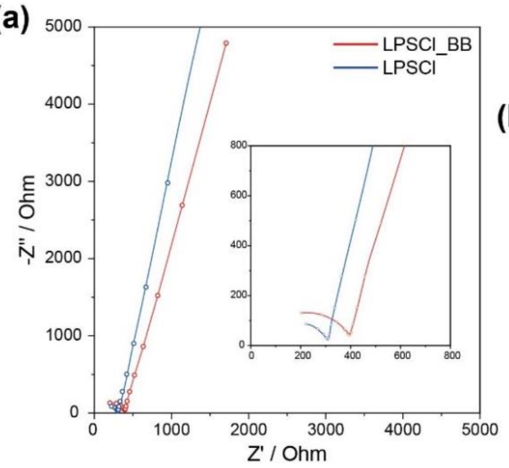

(b)

Electrolyte	Thickness of pellet (um)	Resistance (Ω)	Ionic conductivity (mS/cm)
LPSCl	725.0	304.9	0.8
LPSCl_BB	713.8	388.2	0.7

Figure S12. Ionic conductivity of solid electrolytes. (a) Electrochemical impedance spectroscopy (EIS) spectra and (b) ionic conductivities of pristine Li_6PS_5Cl (LPSCl) and LPSCl after exposure to butyl butyrate solvent at 25°C.

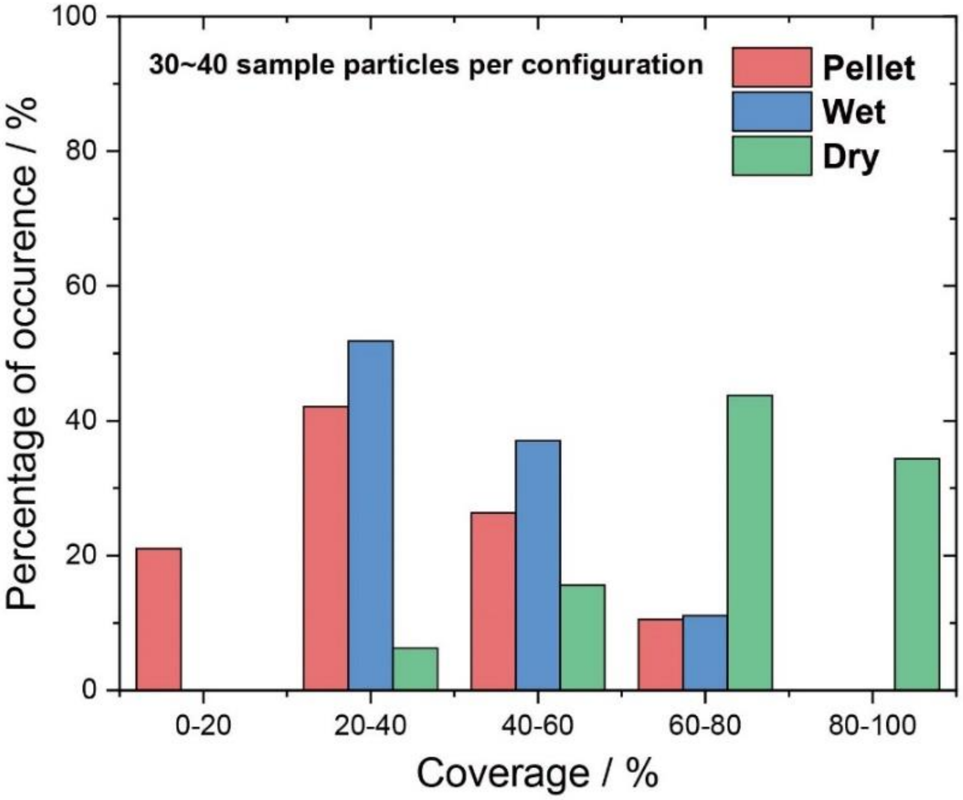

Figure S13. Coverage distribution in electrode configurations. Distribution of sample particle coverages in pellet, wet-processed, and dry-processed electrodes.

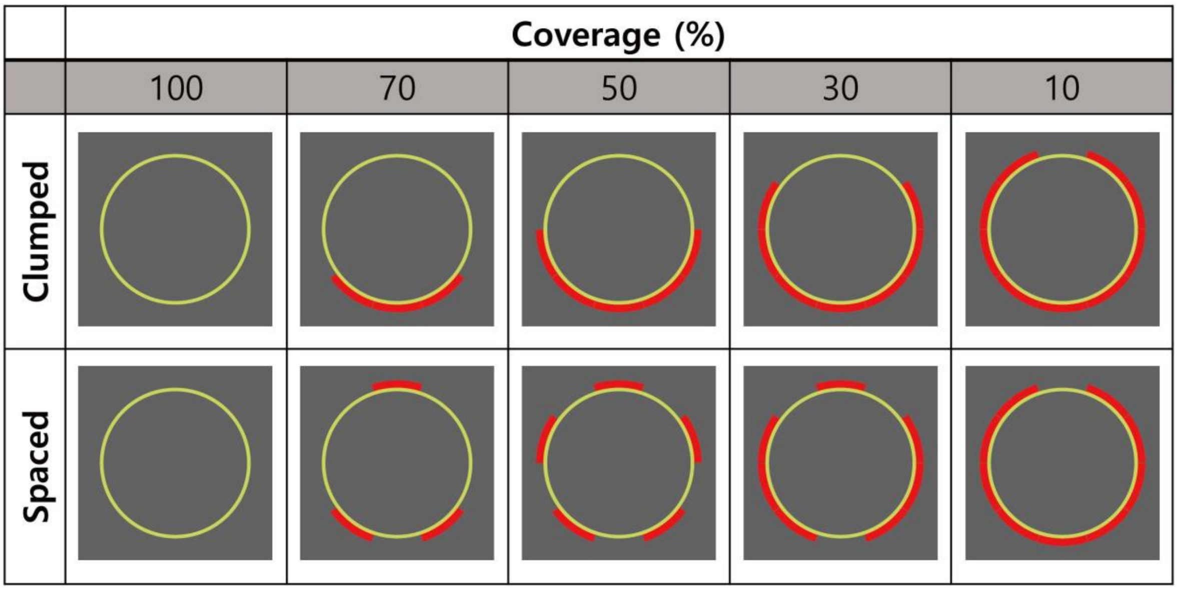

Figure S14. Different types of coverages. Schematic illustration of clumped or spaced particles with different coverages.

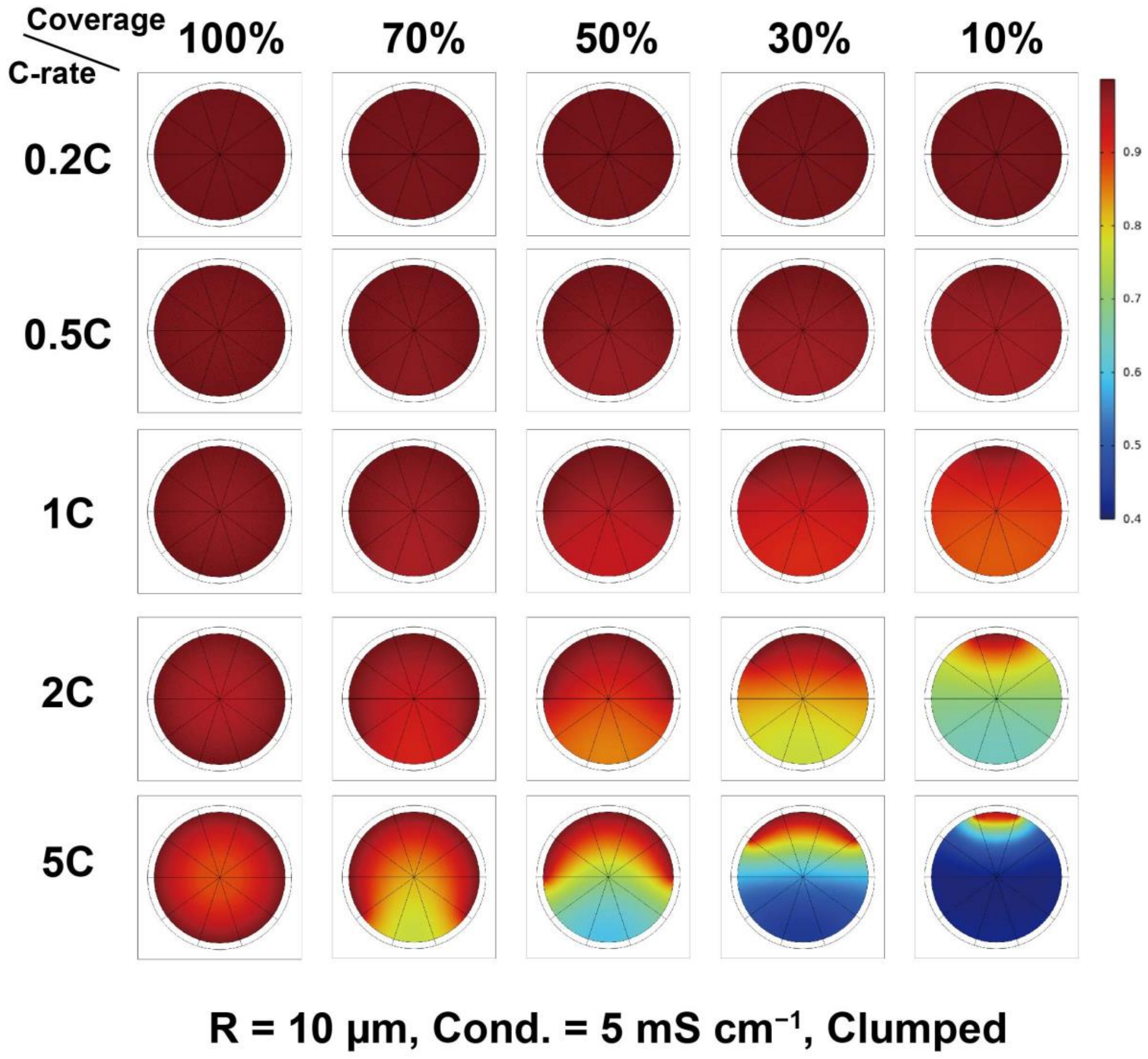

Figure S15. Simulation results with enhanced conductivity. Electrochemical modeling results of single lithium nickel manganese cobalt oxide (NMC) particle with different coverages and C-rates. Lithium-ion concentration contours in the NMC particles at the end of discharging. The modelling parameters: R = 10 μm, cond. = 5 mS cm^{-1}, and clumped configuration, where R indicates the radius of the particle and cond. indicates the ionic conductivity of solid electrolytes.

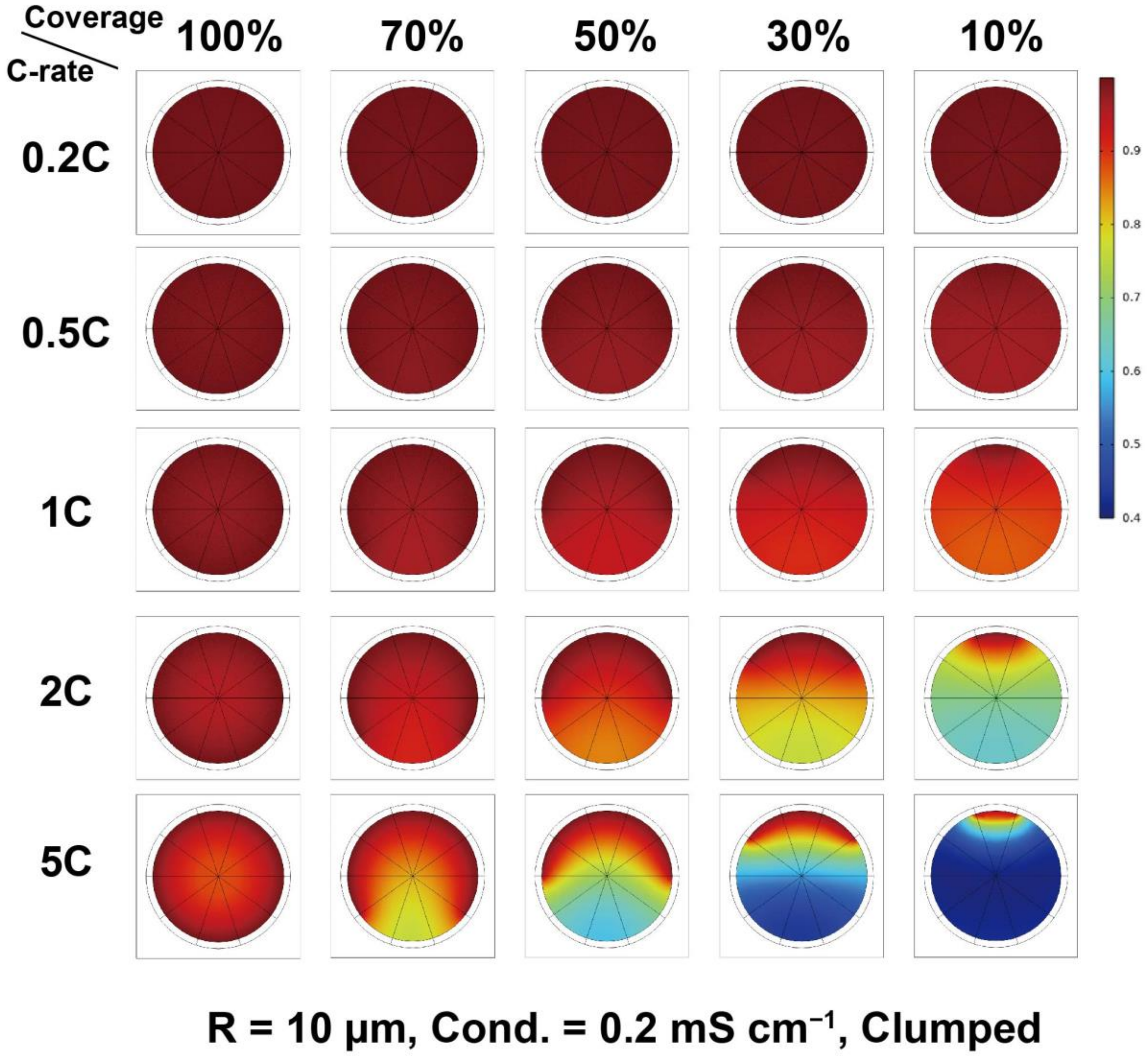

Figure S16. Simulation results with reduced conductivity. Electrochemical modeling results of single lithium nickel manganese cobalt oxide (NMC) particle with different coverages and C-rates. Lithium-ion concentration contours in the NMC particles at the end of discharging. The modelling parameters: R = 10 μm, cond. = 0.2 mS cm^{-1}, and clumped configuration, where R indicates the radius of the particle and cond. indicates the ionic conductivity of solid electrolytes.

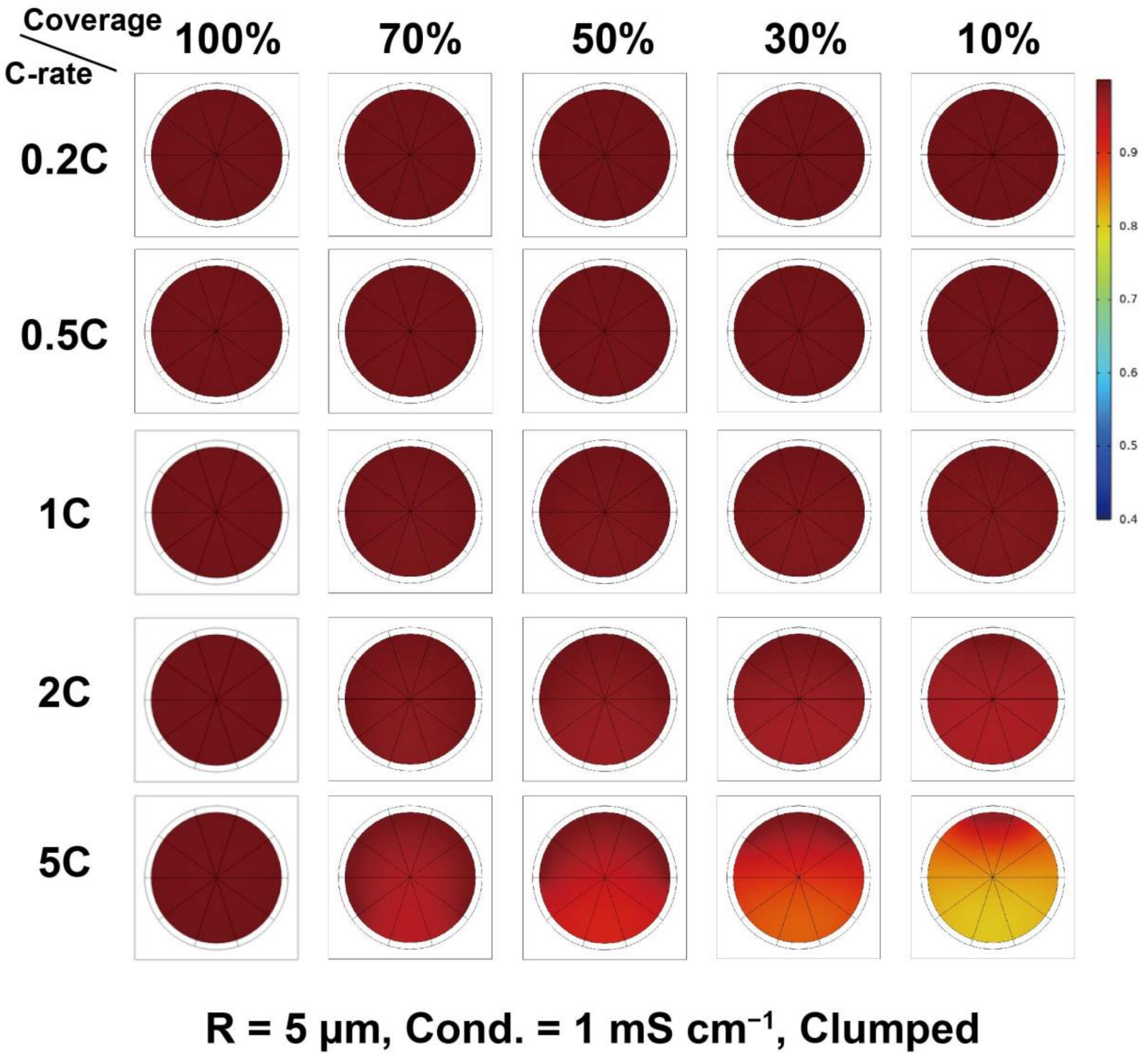

Figure S17. Simulation results with medium particle size. Electrochemical modeling results of single lithium nickel manganese cobalt oxide (NMC) particle with different coverages and C-rates. Lithium-ion concentration contours in the NMC particles at the end of discharging. The modelling parameters: R = 5 μm, cond. = 1 mS cm^{-1}, and clumped configuration, where R indicates the radius of the particle and cond. indicates the ionic conductivity of solid electrolytes.

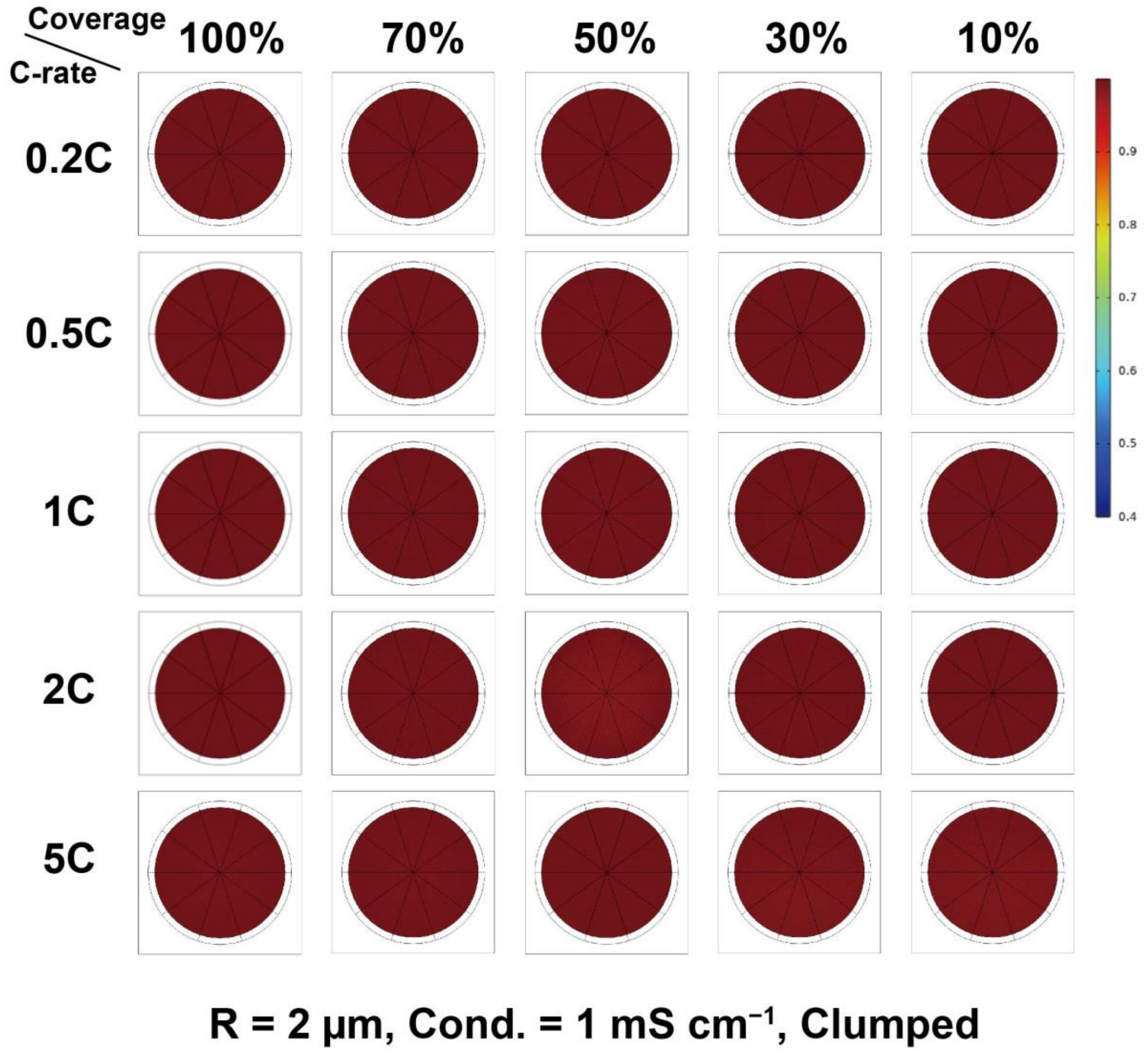

Figure S18. Simulation results with small particle size. Electrochemical modeling results of single lithium nickel manganese cobalt oxide (NMC) particle with different coverages and C-rates. The modelling parameters: R = 2 μm, cond. = 1 mS cm^{-1}, and clumped configuration, where R indicates the radius of the particle and cond. indicates the ionic conductivity of solid electrolytes.

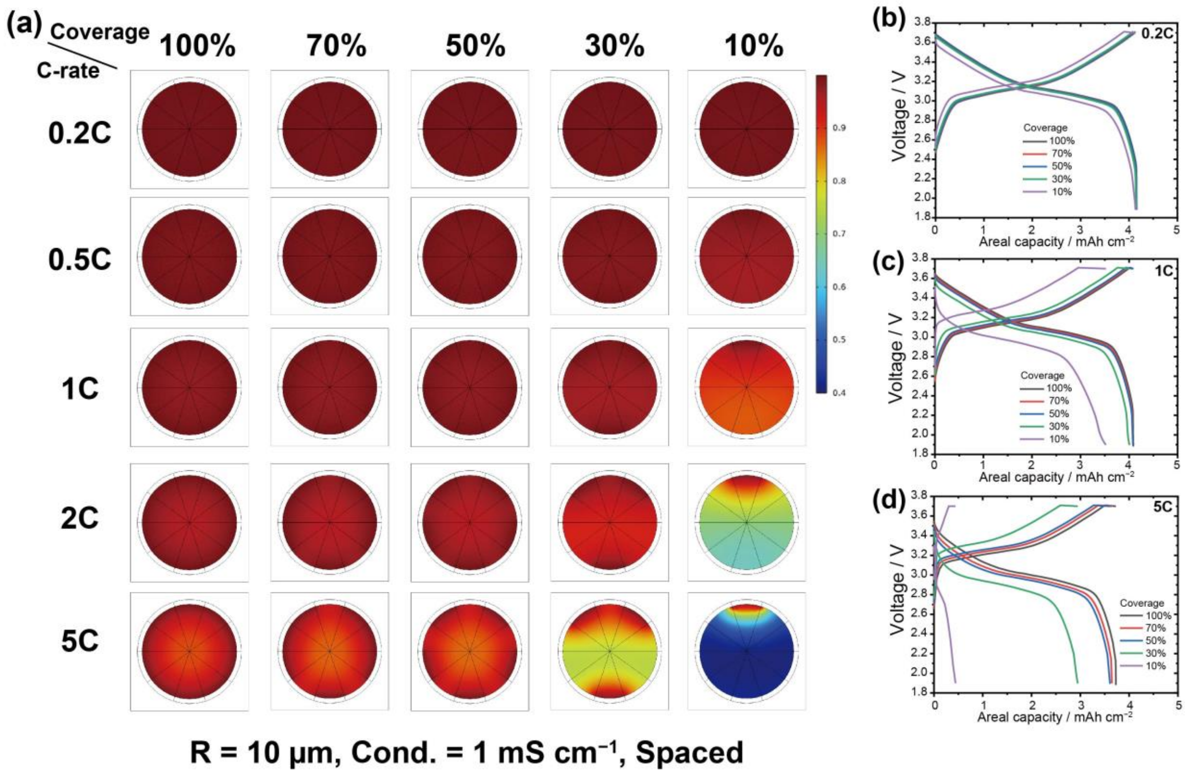

Figure S19. Simulation results spaced coverages. Electrochemical modeling results of single lithium nickel manganese cobalt oxide (NMC) particle with different coverages and C-rates. (a) Lithium-ion concentration contours in the NMC particles at the end of discharging. Voltage profiles at different C-rates of (b) 0.2C, (c) 1C, and (d) 5C. The modelling parameters: R = 10 μm, cond. = 1 mS cm^{-1}, and spaced configuration, where R indicates the radius of the particle and cond. indicates the ionic conductivity of solid electrolytes.

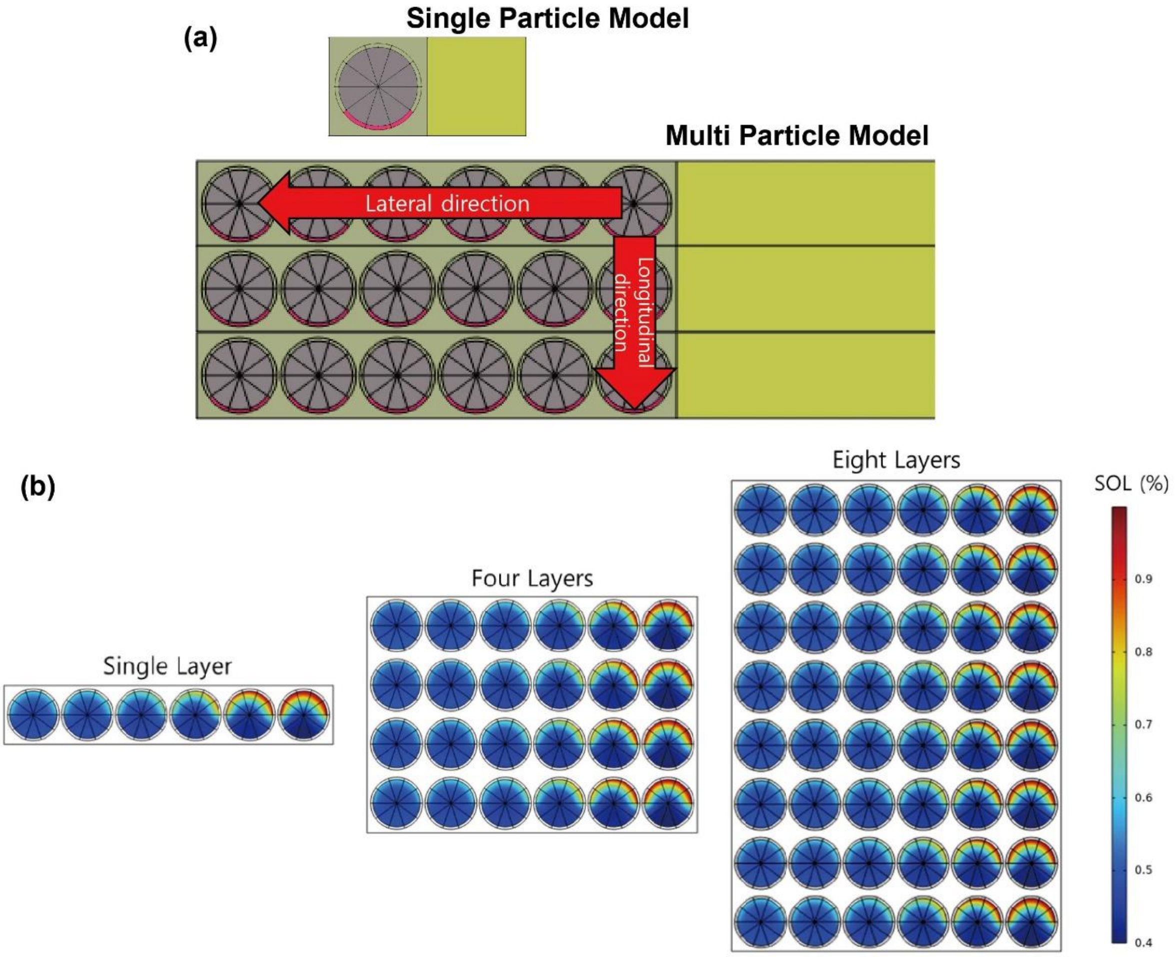

Figure S20. Expansion to multi-particle model. (a) Schematics of single-particle and multi-particle models. (b) Lithium-ion concentration contours of multi-particle models at the end of discharge, depending on longitudinal direction.

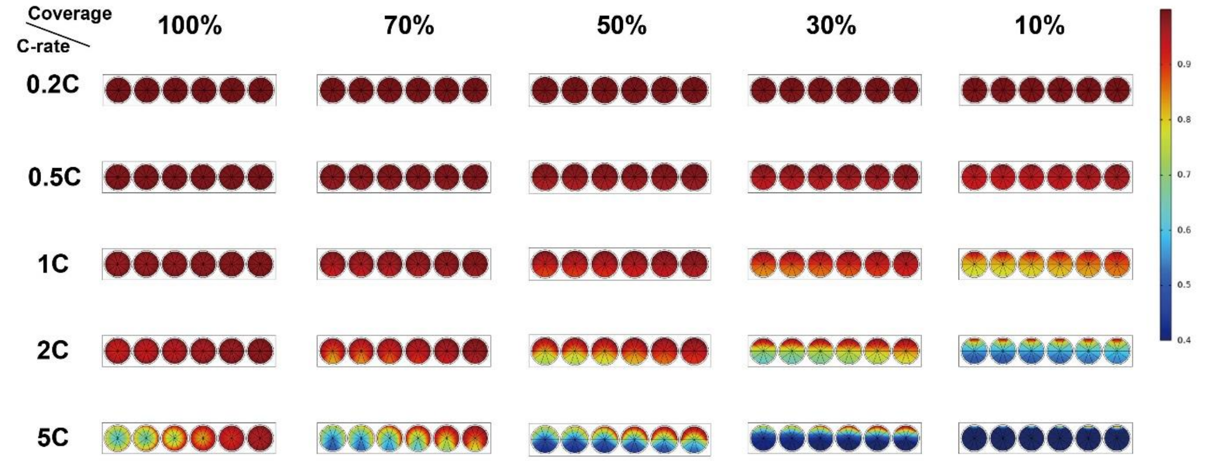

Figure S21. Simulation results with multiple particles. Lithium-ion concentration contours at the end of discharge of multi-particle models with different coverages, C-rates, and same orientations.

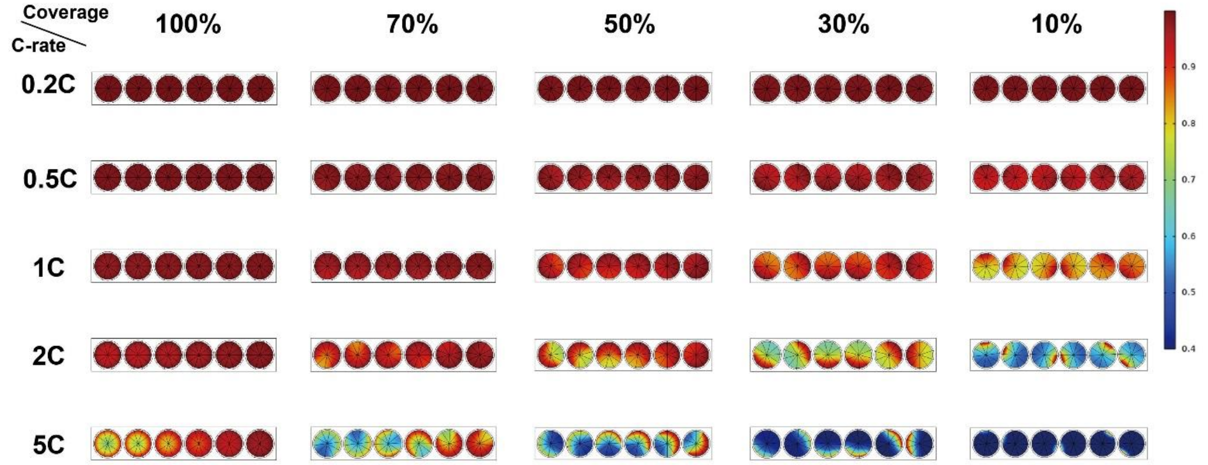

Figure S22. Simulation results random orientations. Lithium-ion concentration contours at the end of discharge of multi-particle models with different coverages, C-rates, and random orientations.

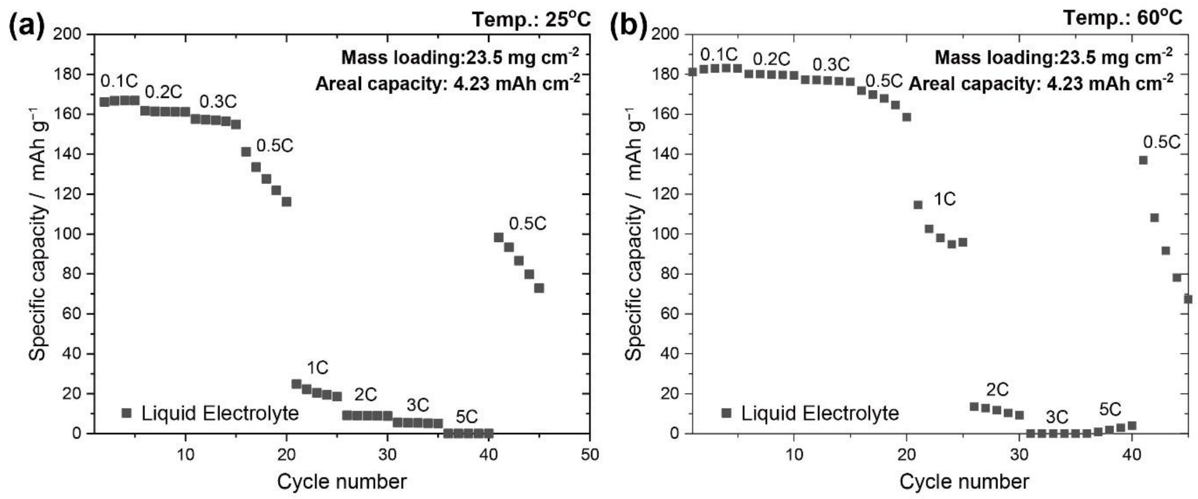

Figure S23. Rate capability tests with liquid electrolyte. Rate capability of cells with liquid electrolyte at (a) 25°C and (b) 60°C. The mass loading and areal capacity were 23.5 mg cm^{-2} and 4.23 mAh cm^{-2}, respectively.

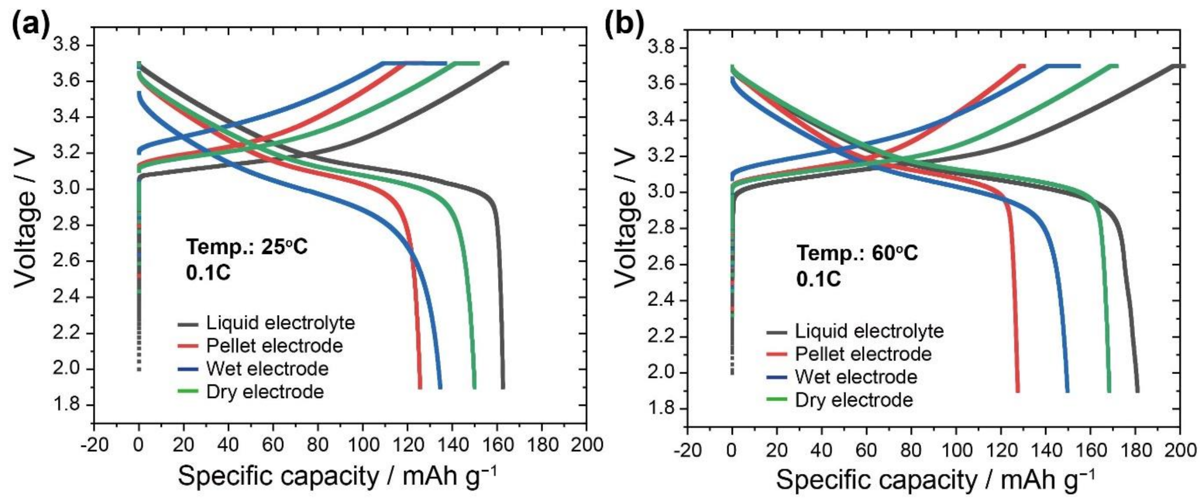

Figure S24. Voltage profiles at 0.1C. Voltage profiles of liquid electrolyte, pellet, wet, and dry electrodes at (a) 25°C and (b) 60°C.

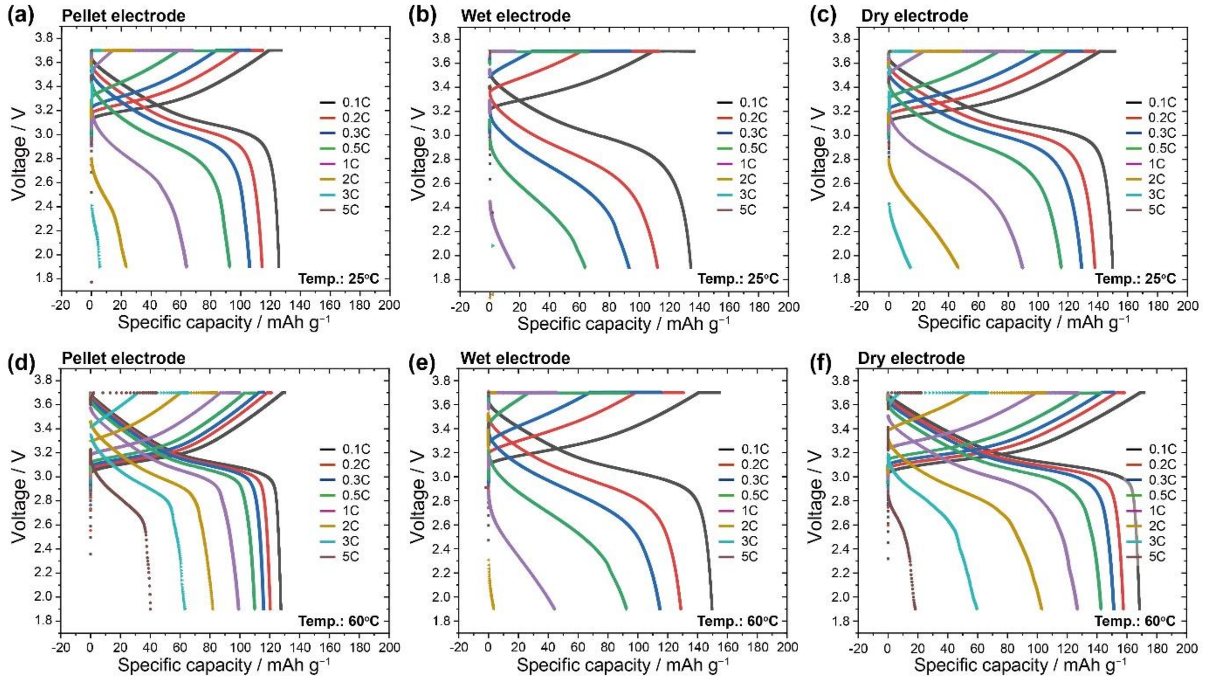

Figure S25. Voltage profiles at different C-rates. Voltage profiles of (a, d) pellet, (b, e) wet, and (e, f) dry electrodes with various C-rates at 25°C and 60°C.

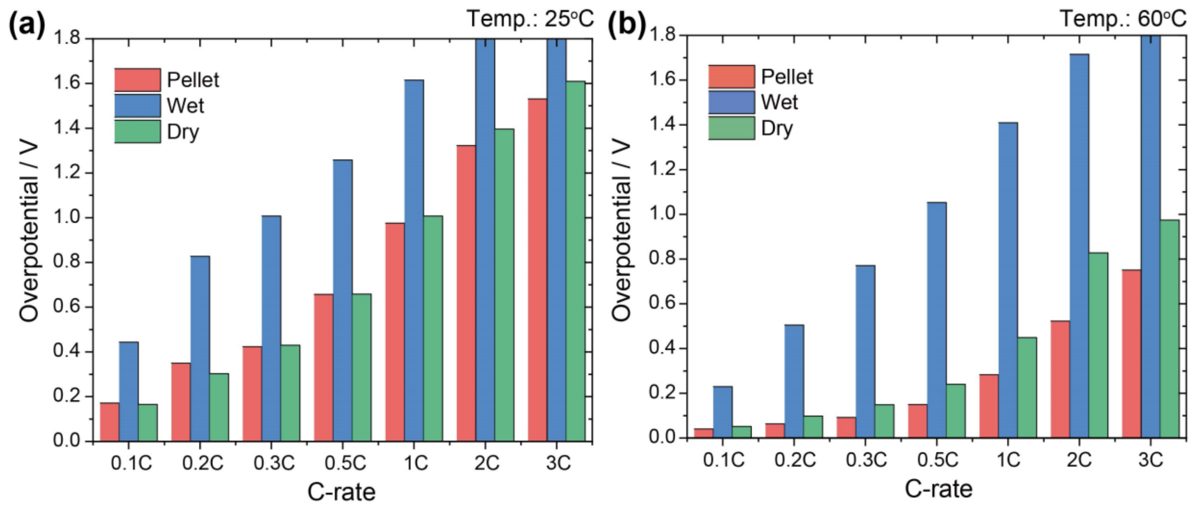

Figure S26. Analysis of overpotential. Overpotential of pellet, wet, and dry electrodes at (a) 25°C and (b) 60°C.

Name	Value	Description	Reference
T	333 [K]	Temperature	tuned
A	1.3265 [cm^2]	Area of cathode	measured
L_pos	25 [μm]	Thickness of cathode	tuned
L_sep	140 [μm]	Thickness of electrolyte	measured
D	36.378 [m]	Depth of cathode	calculated
rp	2 ~ 10 [μm]	Particle radius	measured
V	11.429 [mm^3]	Volume of active materials	calculated
sigma_l	0.2 ~ 5 [mS/cm]	Ion conductivity of electrolyte	Reference [2]
sigma_b	0.1 ~ 30 [S/m]	Electrical conductivity of conductive binder domain	Reference [3]
i0_pos	2 [A/m^2]	Reference exchange current density of cathode (at room temperature)	Reference [4]
i0_neg	100 [A/m^2]	Reference exchange current density of anode	tuned
cs0_pos	47,521 [mol/m^3]	Initial ion concentration in cathode	tuned
i_1C	5.44 [mA]	Applied 1C current	calculated
C_dl	0.2 [F/m^2]	Electrical double layer capacitance	tuned

Table S1. Modeling parameters. Parameter ranges used for various modeling.

References

1. Chaouachi O, Réty J-M, Génies S, Chandesris M, Bultel Y. Experimental and theoretical investigation of Li-ion battery active materials properties: Application to a graphite/Ni0.6Mn0.2Co0.2O2 system. *Electrochimica Acta* **366**, 137428 (2021).

2. Kraft MA*, et al.* Influence of Lattice Polarizability on the Ionic Conductivity in the Lithium Superionic Argyrodites Li6PS5X (X = Cl, Br, I). *Journal of the American Chemical Society* **139**, 10909-10918 (2017).

3. Pantea D, Darmstadt H, Kaliaguine S, Sümmchen L, Roy C. Electrical conductivity of thermal carbon blacks: Influence of surface chemistry. *Carbon* **39**, 1147-1158 (2001).

4. Tsai P-C*, et al.* Single-particle measurements of electrochemical kinetics in NMC and NCA cathodes for Li-ion batteries. *Energy & Environmental Science* **11**, 860-871 (2018).
